# Study protocol for a comparative effectiveness trial of two parent training programs in a fee-for-service mental health clinic: can we improve mental health services to low-income families?

**DOI:** 10.1186/1745-6215-15-70

**Published:** 2014-03-01

**Authors:** Deborah A Gross, Harolyn ME Belcher, Mirian E Ofonedu, Susan Breitenstein, Kevin D Frick, Budhathoki Chakra

**Affiliations:** 1 Johns Hopkins University School of Nursing, 525 North Wolfe Street, Baltimore, MD 21205, USA; 2 Kennedy Krieger Institute, 1750 E. Fairmont Avenue, Baltimore, MD 21231, USA; 3 Rush University College of Nursing, 600 S. Paulina Avenue, Chicago, IL 60612, USA; 4 Johns Hopkins University Carey School of Business, 100 International Drive, Baltimore, MD 21202, USA

**Keywords:** Child mental health, Comparative effectiveness research, Parent training, Implementation in fee-for-service clinic, Preschool behavior problems, Chicago Parent Program, Parent-Child Interaction Therapy, Cost

## Abstract

**Background:**

Untreated behavioral and mental health problems beginning in early childhood are costly problems affecting the long-term health and wellbeing of children, their families, and society. Although parent training (PT) programs have been demonstrated to be a cost-effective intervention modality for treating childhood behavior problems, they have been less effective for children from low-income and underserved racial and ethnic populations. The purpose of this randomized trial is to compare the effectiveness, cost, and social validity of two manualized evidence-based PT programs that were developed and tested on different populations and employ different delivery models: (1) The Chicago Parent Program (CPP), a group-based program developed in collaboration with a community advisory board of African-American and Latino parents; and (2) Parent-Child Interaction Therapy (PCIT), an individualized parent-child coaching model considered to be ‘the gold standard’ for parents of children with externalizing behavior problems.

**Methods:**

This trial uses an experimental design with randomization of parents seeking behavioral treatment for their 2- to 5-year-old children at a mental health clinic in Baltimore, MD (80% African-American or multi-racial; 97% receiving Medicaid). Using block randomization procedures, 262 parents are randomized to CPP or PCIT. Clinicians (n = 13) employed in the mental health clinic and trained in CPP or PCIT are also recruited to participate. Primary outcomes of interest are reductions in child behavior problems, improvements in parenting, perceived value of the interventions from the perspective of parents and clinicians, and cost. Parent distress and family social risk are assessed as modifiers of treatment effectiveness. We hypothesize that CPP will be at least as effective as PCIT for reducing child behavior problems and improving parenting but the programs will differ on cost and their social validity as perceived by parents and clinicians.

**Discussion:**

This is the first study to compare the effectiveness of a PT program originally designed with and for parents from underserved racial and ethnic populations (CPP) against a well-established program considered to be the ‘the gold standard’ (PCIT) with a high-risk population of parents. Challenges related to conducting a randomized trial in a fee-for-service mental health clinic serving urban, low-income families are discussed.

**Trial registration:**

NCT01517867

## Background

There are over 20 million children in the United States under the age of 5 years and 10% to 30% are estimated to have significant behavior problems affecting their social relationships and abilities to succeed in school [1,2]. Young children with high rates of behavior problems are more likely to be expelled from preschool, struggle with academic expectations, and have difficulty with peer relationships [3,4]. Persistent conduct problems beginning early in life are the most potent predictor of later antisocial behavior [5,6]. Over time, annual public costs for untreated child behavior problems can range from US$24,000 to over US$61,000 per child for mental health services, grade retention, special education, and juvenile justice involvement [7,8]. Long-term, behavioral and mental health problems during childhood have a stronger impact on children’s future productivity and earnings potential than do physical health problems [9]. In sum, untreated child behavior problems are costly problems affecting the long-term health and wellbeing of the child, their family, and society.

Parent training (PT) is a well-researched, cost-effective intervention modality for treating behavior problems in early childhood by teaching parents effective child management techniques [10]. Meta-analyses show that one-third to two-thirds of children with diagnosed conduct problems (for example, oppositional defiant disorder, ADHD) demonstrate clinically significant improvements following PT [11]. However, most of the evidence-based PT programs available were originally developed and tested on middle-class White families [12,13]. Overall, the strongest PT outcomes are found among more economically advantaged and White families. It is unclear whether poorer outcomes among low-income children and children of color are due to differences in access to treatment, perceived stigma associated with mental health treatment, acceptability of treatment models, or other factors. To improve PT outcomes, adaptations to program protocols have been applied for specific underserved populations [13,14]. However, there is little empirical evidence that many of these modified programs are effective or represent valid adaptations of the original treatment. Importantly, the field continues to struggle with the challenge of providing evidence-based treatments that engage and benefit low-income children and families from underserved racial and ethnic populations [15].

### Study purpose

This randomized trial will compare the effectiveness, cost, and social validity of two evidence-based parenting skills programs: Parent-Child Interaction Therapy (PCIT) and the Chicago Parent Program (CPP). PCIT and CPP were developed to improve parenting quality and reduce behavior problems in young children aged 2 to 5 years. Both PT programs teach essentially the same core behavioral management strategies [16], are listed on the National Registry of Evidence-based Prevention Programs (see http://www.nrepp.samhsa.gov), and rely on similar social learning theories for changing parent and child behaviors [17]. However, they differ markedly in their delivery models; the target populations they were originally designed to help; treatment duration; and requirements for clinician qualifications, training, and certification (see Table 1). These differences have significant implications for the cost, implementation burden for mental health agencies, and effectiveness of mental health treatments targeting young children from low-income communities. Consistent with the goals of comparative effectiveness research [18], the purpose of this study is to compare the benefits, perceived value, and cost of two alternative mental health treatments for young children. The long-term goal is to assist parents, clinicians, administrators, and policy makers to make informed decisions that will improve mental healthcare of young children from low-income families.

**Table 1:** Comparison of Parent-Child Interaction Therapy (PCIT) and Chicago Parent Program (CPP) populations, delivery models, clinician training, and fidelity procedures

**Parent training variable**	**PCIT**	**CPP**
Original validation sample	Children aged 2 to 7 years with externalizing behavior disorders	Children aged 2 to 5 years from community samples
	Predominantly White, lower/middle and middle income families	Predominantly African-American and Latino, low-income families
Delivery format	Live, individual coaching of parent-child dyad	Parent groups
Treatment participants	Parent, child, one PCIT therapist	Eight to 12 parents, two CPP group leaders, no children
Treatment components	Manualized program	Manualized program
	Therapist provides didactic and live skills coaching sessions	Parents watch/critique video vignettes of parent-child models
	Parent wears microphone in their ear to receive clinician feedback and support	Clinician-led parent group discussions, role play exercises, and group activities
	Weekly homework	Weekly homework
	Progress based on mastery attainment	Progress based on session attendance
Parenting skills taught	Child-directed interaction skills	Child-directed interaction skills
	Child management skills	Child management skills
	Stress management	Stress management
	Problem-solving skills	Problem-solving skills
Clinician qualifications	Masters degree or higher	High school diploma or higher
	Licensed mental health service provider (or 4th year doctoral student in psychology)	Outstanding interpersonal skills (based on references)
		Prior experience working with parents
Basic clinician training	Five-day workshop (40 hours)	Two-day workshop (16 hours)
Clinician certification	Completion of (1) initial training, (2) continued training, (3) certified PCIT therapist application. Certification renewable every 2 years	Completion of (1) initial training workshop, (2) pass written test, (3) lead at least two CPP groups, (4) three fidelity assessments exhibiting adherence and skill in conducting CPP groups
Fidelity monitoring procedures	Independent fidelity assessments,	Independent fidelity assessments
	Submit video-recorded PCIT sessions	Submit audio-recorded CPP sessions

### Parent-Child Interaction Therapy (PCIT)

PCIT was developed in the early 1970s and initially validated in studies comprised mainly of White, two-parent families of children with behavior problems. Its effectiveness has been supported by over two decades of research [19,20] and it is widely considered to be ‘the gold standard’ among PT therapies for children with significant conduct problems.

There are two phases to PCIT: child-directed interaction and parent-directed interaction. During the child-directed interaction, parents are taught to follow the child’s lead during play and coached to use skills such as praise, encouragement, and descriptive commenting (that is, describing aloud what the child is doing so as to teach and engage the child without controlling the interaction). This phase of treatment is intended to build the parent-child relationship and create a foundation of positive experiences for the child and parent. Once parents’ skill levels meet a predetermined set of mastery criteria (based on clinician observation during the PCIT session), they begin the phase of parent-directed interaction which focuses on child behavior management. In this second phase, parents are taught to provide clear, direct commands, and to follow through on commands using consequences for compliance (for example, praise) and non-compliance (for example, time-out). Parents’ progress is assessed at each session and treatment progression is guided by the rate at which parents master each new set of skills. Parents are assigned weekly ‘homework’ intended to help them practice the new skills at home with their children and achieve mastery.

Treatment progress is monitored weekly using parent-rated and clinician-rated assessments of child behavior. According to published research, most families complete the full course of treatment in 10 to 20 weekly, 1-hour clinic-based sessions [21]. However, clinicians using PCIT with low-income families from underserved racial and ethnic populations report longer courses of treatment that can extend up to 8 months to achieve mastery of PCIT-specific parenting skills [22].

A unique aspect of PCIT is the way parents are coached during the treatment sessions. During the sessions, parents wear a microphone-in-the-ear hearing device and are guided on their use of the skills by a clinician who is observing the parent-child interaction from behind a one-way mirror. This strategy requires that clinical facilities have a one-way mirror and audio equipment, and that clinicians are highly trained to coach parents in accordance with the protocol. Therapists trained in PCIT attend a 5-day workshop and receive additional training and fidelity monitoring. PCIT certification may take 6 to 12 months to complete depending on the number of families and the proficiency of the clinician.

There is extensive research demonstrating that PCIT has led to substantial improvements in parent and child behavior up to several years post-treatment. Effect size (*d*) behavioral improvements among families who complete PCIT have ranged from 0.61 to 5.67 [20,23]. However, many parents prematurely stop attending PCIT, particularly parents from low income and under-represented racial and ethnic backgrounds. In this population, PCIT attrition ranges from 56% to 67% of families [24,25]. Indeed, the strongest predictor of attrition in PCIT appears to be low family income and the most common reason cited by parents for the attrition is ‘disagreement with the treatment approach; [26], p. 436. As a result, it is difficult to determine the effectiveness of PCIT in parents from low-income and under-represented racial and ethnic backgrounds.

### The Chicago Parent Program (CPP)

The CPP was developed in 2002 specifically to address the gap in evidence-based PT programs for parents from underserved racial and ethnic populations raising young children in low-income communities [27]. It is delivered in 12 weekly 2-hour parent group sessions with the first 4 weeks focused on building a positive relationship with the child and the second 4 weeks focused on child behavior management. The last 4 weeks center on stress management, problem-solving skills, and skill maintenance, concepts interwoven into PCIT individual sessions. Groups are led by two trained leaders and are typically comprised of eight to 12 parents. Children do not participate in the group sessions and, therefore, childcare needs to be provided during CPP sessions. Refreshments are provided for parent group members, children, and staff supervising the children.

CPP is a manualized program that uses a combination of video vignettes and parent group discussion to teach behavioral management techniques. These vignettes show parents interacting with their children and managing misbehavior in a range of situations at home and in public places; most vignettes depict families from underrepresented racial and ethnic groups. Parents can watch and critique parent-child models, guided by the group leader discussion questions listed in the CPP Group Leader Manual. Weekly ‘homework’ is assigned to help parents practice the new skills at home with their children.

There are several unique features of CPP. Because it was designed to be culturally and contextually relevant for parents from under-represented racial and ethnic populations living in low-income urban neighborhoods, some parenting strategies common in all PT programs are described or managed differently in CPP. For example, parent-child play, a centerpiece of most PT programs including PCIT, is not highlighted as an essential strategy for building a good relationship with their child. This is because advisory board parents considered parent-child play to be a somewhat frivolous activity valued by middle-class parents with leisure time [28]. Instead, CPP teaches parents about ‘child-centered time’ , defined as time parents spend with their children focused on the child’s interests but which could occur in a variety of non-play situations (for example, cooking together, grocery shopping, bath time). Other modifications based on parent advisory board input include those related to spanking, time-outs, and family routines and traditions (see Gross et al. [28] for more detail on the CPP advisory board input).

Unlike PCIT, parents do not practice the new skills with their children during the treatment session and there are no opportunities for CPP group leaders to directly observe or critique how parents are implementing the new skills with their children. Instead, role-play exercises are built into each CPP session allowing parents to practice the skills with other parents under the guidance of the group leader. In addition, weekly ‘homework’ is assigned at the end of the group session and reviewed at the beginning of the following group session in order to assess parent enactment of the skills and child response.

CPP group leaders complete a 2-day training workshop. CPP group leaders can become certified, which requires submission of three or more audio recorded group sessions demonstrating high levels of adherence and competence as assessed by independent raters using the Chicago Parent Program Fidelity Checklist [29].

Evaluated in multiple controlled trials, the CPP has led to significant improvements in children’s behavior problems and parenting up to 1 year post intervention [30]. Over 90% of participants in these studies were low-income and from under-represented racial and ethnic groups. However, unlike PCIT, previous randomized trials of CPP have not targeted children with behavior disorders; all studies focused on prevention in community populations. Although average participation rates among parents in these studies was 67% of CPP sessions, approximately one-third of parents never attended any CPP sessions. Parents who do not attend a CPP session miss the content delivered in that session; there are no make-up sessions. The most common reason given by parents for non-attendance is having too many other demands or responsibilities [31].

## Methods/Design

### Study design

This study uses an experimental design with randomization of parents seeking behavioral treatment for their 2- to 5-year old children at a mental health clinic in Baltimore, MD. Equivalence or non-inferiority studies are used to determine whether a novel treatment or health delivery model, that might be less costly or more acceptable to the treatment population, is at least as effective as another more established treatment [32]. In addition to effectiveness and cost, we are comparing the social validity of these two PT program as assessed by the clinicians delivering the program and parents being served since client and practitioner acceptance is necessary for effective implementation. Finally, subgroup analyses will be conducted to identify groups that would most and least benefit from each PT intervention based on baseline child behavior problems scores and degree of family social risk.

A modified intent-to-treat design will be used. In a superiority trial, all randomized participants, regardless of whether they attended any PT sessions, would be included in analyses using an intent-to-treat design. However, such analyses reduce statistical power, increase the chance of finding no differences, and threaten the validity of results in equivalence and non-inferiority studies [33]. Conversely, a per protocol analysis, which would include only parents who attend all recommended sessions, would diminish external validity based on research showing that low attendance rates are common among economically disadvantaged treatment populations [15,34]. Therefore, in this study, participants who attend at least one PT session are included in the analyses and in estimates of attrition. A dose-response analysis will be conducted to understand the effects of attendance and hours of treatment exposure on outcomes.

This study was first reviewed and approved by the Institutional Review Board of the Johns Hopkins University School of Medicine on 7 September 2010. A Certificate of Confidentiality was obtained on 26 July 2011.

### Sampling design: parent-child dyads

The trial setting is a large mental health clinic located in Baltimore, MD. Approximately 80% of the clinic population is African-American or multi-racial and 97% receives Medicaid. Based on a power analysis, we are recruiting 262 parent-child dyads over 5 years (131/condition; allowing 20% attrition). A block randomization procedure is being used to facilitate the initiation of CPP groups without lengthy waiting periods. Each block consists of 10 parent-child dyads.

Inclusion criteria are: (1) ‘parent’ is the biological or adoptive parent or the legal guardian for the target child; (2) parent is English-speaking; (3) child is aged 2 to 5 years; (4) child is brought into treatment by the parent because of externalizing (for example, aggression, inattention) or internalizing (for example,, anxiety, depression) behavior problems; and (5) parent is willing to be randomly assigned to PCIT or CPP. Exclusion criteria are: (1) parent has severe mental illness that would interfere with their ability to participate in PT; (2) parent is actively using drugs or alcohol, based on clinician assessment at intake; and (3) parent has significant cognitive impairment that would affect their ability to understand the PT information presented. Child exclusion criteria include: (1) actively suicidal, psychotic, or have significant developmental delay; (2) congenital, genetic, or sensory anomalies that would interfere with participating in PT; and (3) diagnosis of autism or pervasive developmental disorder. Excluded children are referred for appropriate treatment services or hospitalization. Only one parent and one child per family are eligible to participate in the study.

### Parent-child recruitment strategy

Parent-child participants are primarily recruited from the population of active patients at the mental health clinic (n = 150 to 180 new referrals per year). Parents of children aged 2 to 5.5 years are informed about the study following their intake and diagnostic interview. The diagnostic interview clarifies the mental health and psychosocial needs of the family and helps to determine whether the parent and child meet study eligibility criteria. Parents who expressed interest in the study are contacted by a research team member to inform them about the details of the study, answer study related questions, and obtain informed consent. Randomization and scheduling to complete parent-child baseline assessment is completed at that time. Additional recruitment is also taking place in the community using flyers posted in early childhood agencies and presentations to providers serving families with young children.

In this study, attrition is defined as a parent who: (1) consents to participate; (2) is randomized to CPP or PCIT; (3) completes baseline assessments; (4) attends at least one PT session; but (5) does not complete any of the follow-up assessments.

### Sampling design and recruitment: mental health clinicians

Study clinicians are recruited from the participating mental health clinic. Criteria for inclusion are: (1) the clinician is employed as a therapist at the participating mental health clinic, (2) the clinician has successfully completed either PCIT or CPP training; (3) the clinician is willing to have their PT sessions recorded and submitted to fidelity monitoring; and (4) the clinician is willing to complete research measures. To minimize contamination, clinicians are assigned only to the PCIT or CPP condition based on their training. To date, we have consented 13 mental health clinicians (9 CPP and 4 PCIT).

### Measures

The primary outcomes of interest are reductions in child behavior problems, improvements in parenting quality, perceived value of the PT treatment from the perspective of parents and clinicians (that is, social validity), and cost. In addition, we will assess indicators of parent distress and family social risk as modifiers of treatment effectiveness.

Child behavior problems will be measured using a parent-report survey and rater observation. The Child Behavior Checklist 1½ -5 (CBCL) is a widely used 99-item parent-report measure of externalizing and internalizing behavior problems in preschool children [35]. The CBCL is a standardized measure with demonstrated reliability and validity across racial and ethnic groups [36]. Child behavior problems will also be assessed by independent observers from video recorded parent-child interactions during a structured play and clean-up situation using the Dyadic Parent-Child Interaction Coding System (DPICS) [37]. The DPICS measures frequencies of observed child non-compliance, destructive behavior, crying, whining, yelling, and negative behavior and affect. Child behavior problems are measured at baseline, post intervention, and 4-month follow-up.

Parenting quality will be assessed over time using multiple indices, including parent discipline strategies, parenting self-efficacy, and parenting behavior. The Parenting Questionnaire [27,38] assesses parents’ use of warmth (22 items; alpha = 0.82), following through on discipline (6 items; alpha = 0.78), and corporal punishment (4 items; alpha = 0.59) on a scale of 1 (almost never) to 5 (very often). We will also measure parenting self-efficacy using the Toddler Care Questionnaire [30,39], a Likert-type measure of the extent to which parents feel confident about managing a range of tasks and behaviors specific to parenting young children (38 items; alpha = 0.94). Finally, frequency of parents’ use of praise, commands, critical statements, and positive or negative affect with their children will be assessed from the video recorded parent-child play and clean-up sessions using the DPICS*.* These measures of parenting quality will be obtained at baseline, post intervention, and 4-month follow-up.

Social validity is measured from the perspective of parents (that is, parent satisfaction with treatment and attendance rates) and clinicians (that is, clinician satisfaction with the treatment session and perception of parents’ engagement in treatment). Post intervention, parents complete an overall Program Satisfaction form assessing the extent to which they believe they and their children’s behavior have improved as a result of the PT program, the extent to which aspects of the PT program were difficult for them to do, and whether they would recommend the PT program to other parents. Clinicians assess the extent to which parents actively engaged in the PT sessions using the 7-item clinician-rated Engagement Form [31]. Alpha reliability of the Engagement Form is 0.89 and validity has been supported by its significant associations with improvements in teachers’ and parents’ ratings of child behavior problems [31] and independent ratings of implementation fidelity [29]. Since parents are unlikely to attend mental health treatments they do not find it helpful, parent attendance at the PT sessions is also documented.

Since we were unable to find a brief, valid measure of clinician satisfaction that was appropriate for this study, we created a 3-item measure for clinicians to complete after each PT session. This brief Clinician Satisfaction Form asks clinicians to rate the extent to which (1) they felt positive about their clinical performance in the session they had just led, (2) they believed the PT session assigned to their client was the best PT intervention for them at this time, and (3) they wished they could have done things differently in the PT session but could not because of the requirements of PT protocol assigned to their client. Since there are no data on the reliability of this measure, we will evaluate each item individually.

Moderators of treatment effectiveness will also be examined. These include baseline indicators of parent distress (that is, depressive symptoms, parenting stress) using the Center for Epidemiologic Studies Depression Scale-Revised (CESD-R) [40] and the Parenting Stress Index Short Form (PSI-SF) [41]. In addition, family social adversity is estimated using a composite of whether the participating parent has been exposed to each of the following 10 parent indicators: less than a high school education, currently unemployed, annual household income below $20,000, living with four or more people in the same home, current or past history of homelessness, aged under 20 years at study entry, aged under 20 years at birth of first child, history of substance abuse, history of domestic violence, and baseline CESD-R score of 16 or higher. Social adversity items are scored as yes/no; scores range from 0 to 10.

### Costs

We will compare the costs of the PT treatments including costs associated with clinician time required to administer PT, administrative time, childcare, food (CPP only), and space. Parent costs will also be assessed including time spent in PT, time missed from work if parent left work early to attend PT, and additional childcare parent may have needed to provide for other children not brought to the clinic. Parents will complete a Weekly Effort Form at each PT session indicating whether they left work early to attend PT (and if so, how early) and whether arrangements were made for childcare.

We anticipate that many participants in this study will require additional therapeutic services beyond PT (for example, individual therapy, medication). Type, amount, and cost of additional therapies provided to the participating parent or child while receiving PT and during the 4 months immediately after completing PT are being collected.

### Data collection procedures

Following consent, parents and children complete baseline measures of child behavior problems and parenting quality. Parents randomized to the CPP condition are informed of the CPP start date; CPP groups meet at the same time each week. Parents randomized to the PCIT condition are contacted by their assigned study clinician to schedule their first PCIT session.

Post-intervention assessments occur after the 12th CPP session (approximately 4 months post baseline) or after the parent and child are discharged from PCIT. These assessments are scheduled only for parents who attended at least one PT session. A second follow-up assessment is scheduled for parents and children in both conditions 4 months after the post-treatment assessment to capture longer-term changes in parents and children. Following each of the three assessment phases, parents receive $30 and the child receives an age-appropriate book valued at $5 or less.

### Treatment fidelity procedures

Both PT programs are manualized with established procedures for monitoring fidelity. Consistent with their established protocols, all CPP groups are audio recorded and all PCIT sessions are video recorded and a random selection of 20% of these treatment sessions are sent to independent raters to assess quality and adherence to protocol.

### Hypotheses and analysis

Initial analyses will examine data for baseline differences, assumptions of normality, and determination of successful randomization. Violations of normality among continuous dependent variables will be addressed by data transformation. Any measures that cannot be transformed successfully will be analyzed using rank-order statistical models rather than the parametric models described below.

#### H1: CPP will be at least as effective as PCIT for reducing child behavior problems based on parent-report and observation

This hypothesis will be analyzed using a one-sided two-sample t-test at alpha level 0.05, along with a 90% two-sided confidence interval or a 95% one-sided confidence bound. If the outcome variable appears to deviate from approximate normal distribution, a Wilcoxon rank-sum test will be used. Dependent measures associated with child behavior problems will include the CBCL and observed child behaviors derived from the DPICS.

#### H2: CPP will be at least as effective as PCIT for improving parenting discipline, self-efficacy, and observed parenting behavior

This hypothesis will be analyzed using similar methods described above in H1. Dependent measures include parent warmth, follow through on discipline, use of corporal punishment, and observed parent behaviors derived from the DPICS.

#### H3: Parents in the CPP condition will (1) attend a higher percent of PT sessions, (2) be more engaged, and (3) report higher satisfaction than parents in PCIT

Percent attendance by treatment will be compared using 2 × 2 contingency tables and a Fisher’s exact test. Two-sample t-tests will be used to test superiority of CPP over PCIT in Engagement Scale scores by condition. Non-parametric tests will be used to compare differences on the Parent Satisfaction Scale items, and also for Engagement Scale scores if the distribution appears to depart from normality.

#### H4: CPP will yield comparable effects but at less cost and greater satisfaction

We will conduct a cost-consequence analysis (CCA) to compare the costs and consequences of PCIT *versus* CPP. The analysis will be conducted from a societal perspective and separated into the parents’ and clinicians’ perspectives. The dollar value assigned to clinician time will be based on their salaries; in sensitivity analyses we will use data from the Bureau of Labor Statistics to apply an average wage for individuals with similar levels of training. To assign a dollar value to parent time, we will use age-sex average wages for individuals from under-represented racial and ethnic backgrounds as suggested by the United States Panel on Cost-Effectiveness in Health and Medicine [42], data that are also available from the Bureau of Labor Statistics. We will calculate the average cost of clinician time only, of both clinician and parent time, and of all costs per parent to bootstrap the difference between PCIT and CPP to ascertain whether the confidence range overlaps with zero (since data are unlikely to be normally distributed). Since some parents wish to meet individually with their child’s therapist in addition to the PT session, costs associated with all additional therapies will be included in the analyses over two time intervals: while parents are engaged in PT (that is, from baseline to post-intervention) and after PT has ended (that is, from post-intervention to the 4-month follow-up).

#### Exploratory aim: Is PCIT more effective than CPP for some families based on severity of baseline child behavior problems, parent distress, or degree of family social risk?

A secondary analysis of Aims 1 and 2 will be conducted, adding baseline CBCL, CESDR, PSI, and family social risk scores as covariates.

### Study limitations

A limitation of the study design is that the two PT treatments, though roughly equal in the number of treatment hours, require different lengths of time to complete. Treatment duration cannot be made identical without also changing how these treatments are delivered - a key aspect of this study. Nonetheless, we acknowledge that differences in lengths of treatment and child maturation during treatment could confound the results. Treatment duration will be added as a covariate in outcome analyses. A second study limitation is the use of a single urban mental health clinic serving predominantly low-income, African-American families, which reduces the generalizability of results to other populations and clinics. Although employing multiple clinics would have enhanced generalizability, it could also have introduced substantial site effects and increased study cost.

## Discussion

### Current challenges

The study team has encountered multiple recruitment and implementation challenges associated with conducting a randomized trial in an urban, fee-for-service mental health clinic. Three key challenges are described below.

#### Parent-child recruitment

Prior to consenting to be in the study, all families seeking mental healthcare at the clinic must complete a lengthy intake, triage, and diagnostic process to: (1) determine the urgency and safety of the child; (2) ensure the family has adequate insurance coverage (100% of the participants to date have Medicaid coverage); and (3) identify risk factors and diagnoses. These procedures can take up to 2 months or more, due in part to scheduling difficulties, missed phone calls, and parents’ having multiple phone number changes or disconnections. The length of the intake and diagnostic process has contributed to substantial attrition in the pool of eligible families that has limited recruitment. A review of overall patient retention for the clinic over the past year revealed that 58% of families with children in the 2- to 5-year age range who initiate the intake referral fail to make it through the diagnostic interview processes to attend their first treatment session. The study team has increased efforts to recruit families from the surrounding community to boost the number of eligible families in the clinic pipeline. These efforts have included targeted recruitment mailings to homes located in the zip codes surrounding the mental health clinic; flyers posted in neighborhood stores and agencies; recruitment at community events attended by families with young children; and meetings with healthcare providers, early childhood educators, and administrators of agencies serving families and young children to market the study. Once a potential study parent calls for an intake, the research coordinator now assists in the intake and scheduling procedures to expedite the process.

#### Clinician productivity expectations

In a fee for service clinic, clinician job evaluations are partially based on their ‘productivity levels’ , defined by the number of treatment sessions completed monthly. As a result, there may be an incentive for clinicians to schedule families for treatment visits beyond those specified by the PT protocol. Although the families served in this population have extensive mental health and psychosocial needs, adding clinic visits beyond those specified by the PT protocol risks confounding the impact of the study treatment on child outcomes. A balance was required between the treatment needs of this vulnerable population of families and the clinicians’ needs to ensure that their productivity targets were met. A strategy was negotiated with study clinicians whereby additional treatment sessions are scheduled based on parent perceived need, in contrast to clinician-directed additional treatment sessions. At the beginning of each PT session, parents now complete a brief form that queries the parent about their desire or need to schedule a separate individual visit with their child’s clinician (checked yes or no). If parents indicate they wish to schedule an additional appointment, the clinician is notified to initiate scheduling. The added costs, frequency with which parents request additional treatment sessions, and reason for the added sessions (for example, problems related to the parent’s or child’s’ mental health, housing concerns, domestic violence) will be analyzed by treatment arm.

#### Parent retention in PT treatment

Low rates of parent engagement in their children’s mental health treatment is a well-established problem; particularly among low-income and under-represented racial and ethnic populations [15,43]. In anticipation of this problem, study participants provide contact information for multiple friends or relatives whom they would consent to be called if the research team is unable to reach the participant through other means. Nonetheless, contact with participants has often been difficult to maintain, largely due to problems related to their economic circumstances and those of their contacts (that is, disconnected phones, change in residence, or homelessness). To minimize barriers to treatment attendance, parents receive phone calls reminding them of their scheduled treatment sessions and are provided free transportation to the clinic using a local cab service. We have also added several new baseline research measures to the protocol to understand the role of select variables on parent attendance in PT. For example, recent studies suggest that parents prone to avoid close interpersonal relationships may have lower attendance since engagement in mental health treatment requires a willingness and ability to develop a close and trusting relationship with the clinician or with other parents in a therapeutic group [44]. To test this hypothesis, we have added a measure of parents’ attachment style to the baseline assessment battery.

To our knowledge, this is the first study to directly compare a parent training program originally designed with and for parents from underrepresented racial and ethnic populations (CPP) and a well-established program considered to be the ‘gold standard’ (PCIT) in a clinic setting serving an urban, high-risk population of parents and young children. Although there have been some significant challenges with respect to implementing a comparative effectiveness trial in a fee-for-service mental health clinic, it is important to test evidence-based programs in these types of settings to understand their true benefit. In this study, the design and procedures were created in collaboration with the mental health clinicians employed in the clinical agency. This strategy helps to inform implementation, enhances compatibility with current practice, and promotes generalizability to real life settings. Our approach is consistent with the intent of comparative effectiveness research, which is to learn what works best in actual practice [18]. As a result, we believe the results of this study will ultimately inform the way we invest healthcare dollars for improving outcomes for young children at elevated risk for social, emotional, and academic problems.

## Trial status

Recruiting participants.

## Competing interests

Under an agreement between Rush University Medical Center and Dr. Deborah Gross, Dr. Gross is entitled to revenue from sales of the Chicago Parent Program described in this article. This arrangement has been reviewed and approved by the Johns Hopkins University in accordance with its conflict of interest policies. Dr. Gross does not draw income from sales of the Chicago Parent Program. A Data and Safety Monitoring Board reviewed this manuscript and found no bias.

## Authors’ contributions

DG is the study PI. She conceived and led the study design and drafted this manuscript. HB is the study Co-PI. She leads the subcontract for the mental health clinic where this trial is being implemented. She contributed to developing the study protocols and assisted in drafting this manuscript. MO is Project Manager. She leads study recruitment and helped draft parts of this manuscript. SB contributed to the development of the fidelity monitoring procedures for CPP and helped draft parts of this manuscript. KF is the health economist on this study. He developed and wrote the cost analysis design and procedures and reviewed this manuscript. CB is the statistician on this study. He wrote portions of the manuscript and made critical contributions to revisions in the data analytic procedures described in this paper. All authors approved the final version of this manuscript.

## Authors’ information

Deborah Gross, DNSc, RN, FAAN, Leonard and Helen Stulman Endowed Chair in Psychiatric and Mental Health Nursing, Johns Hopkins University School of Nursing and School of Medicine (Department of Psychiatry and Behavioral Sciences). Harolyn M.E. Belcher, MD, MHS, Director of Research, The Family Center at Kennedy Krieger Institute and Associate Professor of Pediatrics, Johns Hopkins University School of Medicine. Mirian E. Ofonedu, PhD, LCSW-C, Research Manager, The Family Center at Kennedy Krieger Institute. Susan Breitenstein, PhD, RN, Assistant Professor, Rush University College of Nursing. Kevin Frick, PhD, Professor and Vice Dean for Education, Johns Hopkins University Carey School of Business. Chakra Buthathoki, PhD, Assistant Professor, Johns Hopkins University School of Nursing.
